# Calculation of Stopping-Power Ratio from Multiple CT Numbers Using Photon-Counting CT System: Two- and Three-Parameter-Fitting Method

**DOI:** 10.3390/s21041215

**Published:** 2021-02-09

**Authors:** Sung Hyun Lee, Naoki Sunaguchi, Akie Nagao, Yoshiyuki Hirano, Hiroshi Sakurai, Yosuke Kano, Masami Torikoshi, Tatsuaki Kanai, Mutsumi Tashiro

**Affiliations:** 1 Heavy Ion Beam Medical Physics and Biology, Graduate School of Medicine, Gunma University, Maebashi 371-8511, Gunma, Japan; tashiro@gunma-u.ac.jp; 2 Graduate School of Medicine, Nagoya University, Nagoya 461-8673, Aichi, Japan; sunaguchi@met.nagoya-u.ac.jp (N.S.); hirano@met.nagoya-u.ac.jp (Y.H.); 3 Department of Electronics and Informatics, Gunma University, Kiryu 376-8515, Gunma, Japan; akie.nagao@gmail.com (A.N.); sakuraih@gunma-u.ac.jp (H.S.); 4 GHMC Group, Accelerator Engineering Corporation, Inage, Chiba 263-0043, Japan; y.kano@aec-beam.co.jp; 5 Gunma University Heavy Ion Medical Center, Gunma University, Maebashi 371-8511, Gunma, Japan; torikoshi@istc.int (M.T.); t.kanai@osaka-himak.or.jp (T.K.); 6 Department of Accelerator and Medical Physics, National Institute of Radiological Sciences, Anagawa Inage-ku, Chiba 263-8555, Japan; 7 Osaka Heavy Ion Therapy Center, Osaka 540-0008, Japan

**Keywords:** photon-counting CT, effective atomic number, electron density, mean excitation energy, stopping-power ratio

## Abstract

The two-parameter-fitting method (PFM) is commonly used to calculate the stopping-power ratio (SPR). This study proposes a new formalism: a three-PFM, which can be used in multiple spectral computed tomography (CT). Using a photon-counting CT system, seven rod-shaped samples of aluminium, graphite, and poly(methyl methacrylate) (PMMA), and four types of biological phantom materials were placed in a water-filled sample holder. The X-ray tube voltage and current were set at 150 kV and 40 μA respectively, and four CT images were obtained at four threshold settings. A semi-empirical correction method that corrects the difference between the CT values from the photon-counting CT images and theoretical values in each spectral region was also introduced. Both the two- and three-PFMs were used to calculate the effective atomic number and electron density from multiple CT numbers. The mean excitation energy was calculated via parameterisation with the effective atomic number, and the SPR was then calculated from the calculated electron density and mean excitation energy. Then, the SPRs from both methods were compared with the theoretical values. To estimate the noise level of the CT numbers obtained from the photon-counting CT, CT numbers, including noise, were simulated to evaluate the robustness of the aforementioned PFMs. For the aluminium and graphite, the maximum relative errors for the SPRs calculated using the two-PFM and three-PFM were 17.1% and 7.1%, respectively. For the PMMA and biological phantom materials, the maximum relative errors for the SPRs calculated using the two-PFM and three-PFM were 5.5% and 2.0%, respectively. It was concluded that the three-PFM, compared with the two-PFM, can yield SPRs that are closer to the theoretical values and is less affected by noise.

## 1. Introduction

An X-ray computed tomography (CT) image is a map of the photon linear attenuation coefficient, which is usually calculated as the sum of the cross-sectional contributions of the photoelectric effect, Compton scattering, and Rayleigh scattering. This linear attenuation coefficient changes as a function of incident X-ray energy and the atomic number of the material [1,2].

A dual-energy CT, where images are taken using different energies, has the advantage that the effective atomic number and the electron density of the material can be estimated, allowing us to know some of the physical properties of the material. This ability to know some of the physical properties of materials is further exploited to calculate the stopping-power ratio (SPR) required to calculate the patient dose in particle therapy, through parameterisation of the effective atomic number and mean excitation energy (*I*-value) [3,4]. However, many studies have reported that dual-energy CT is strongly affected by the beam-hardening effect because the lower-energy X-rays in the polychromatic X-ray beam are more greatly attenuated than the higher-energy X-rays [5,6,7]. Thus, some projections are hardened, such that the amount of detection can vary because of changes in the average X-ray energy [4,8,9,10]. In this case, beam-hardening artefacts in CT images directly affect the effective atomic number and electron density calculation.

Photon-counting CT is a novel alternative approach that counts individual X-ray photons from multiple energy bins. With a single exposure, the detector acquires simultaneous measurements of the photon flux above one or more user-defined energy thresholds. The potential advantage of the photon-counting detector is that the ability to resolve energies enables energy-selective imaging with a single X-ray exposure [5,11]. The flux data obtained for a set of non-overlapping energy windows from a photon-counting CT system can provide information about the energy dependence of the attenuation coefficients of the materials in an object and, therefore, the types of materials present. One of the advantages of photon-counting imaging is that it can reduce beam-hardening artefacts, because the energy-bin images are reconstructed from data acquired via a narrow polyenergetic spectrum, and the energy-bin data are weighed and combined after log normalisation [12]. Although photon-counting CT systems have many theoretical advantages, there are several experimental problems that must be resolved before clinical implementation can begin, such as detector non-uniformity, inter-pixel charge sharing, and poor energy and image resolution after reconstruction [5,13,14]. Photon-counting CT enables multiple-energy X-ray imaging, also called spectral X-ray imaging, which uses more than two energies. The additional number of energy measurements (i.e., bins) provides more discrete information about the transmitted spectra and enables the use of new approaches for the application of material decomposition and tissue differentiation, such as K-edge imaging, and for improving diagnostic accuracy on contrast agents [11,15,16].

SPR is a physical property that can be estimated from dual-energy CT or spectral X-ray imaging by calculating the effective atomic density and electron density for each pixel of the CT image. Simard et al. [17] recently stated that the SPR estimated from spectral X-ray is slightly superior to that estimated from dual-energy CT. The SPR can be measured directly by making a particle beam vertically incident on a material whose physical thickness is known and then, calculating the difference in the thickness of passage in water with or without the material. However, because it is not possible to measure all substances in the human body directly, the method of converting a CT number to the SPR using the CT-number–SPR conversion curve is mainly used in the clinic, but this method is known to have an error of up to 3% [18]. Therefore, the method of estimating the SPR by calculating the effective atomic number and electron density using dual-energy or spectral CT has recently been highlighted because it can estimate the SPR for each pixel of the CT image with a certain level of accuracy. From dual-energy CT images, Rutherford et al. [1] first calculated the effective atomic number and electron density using a three-parameter-fitting method (PFM). Three-PFM is a method of approximating the atomic cross-section to calculate the linear attenuation coefficient with three terms, namely photoelectric attenuation, incoherent scattering, and coherent scattering, and calculating the parameters of each term to fit the theoretical value. However, their three-PFM has not yet been used to calculate the effective atomic number and electron density from multiple CT images, that is, more than two CT images, and the SPR has not yet been calculated from multiple CT images. On the other hand, the two-PFM proposed by Torikoshi et al. [19] has been used more widely because of its mathematical simplicity. Two-PFM is a method of approximating the atomic cross section to calculate the linear attenuation coefficient with two terms, namely photoelectric absorption and combined Compton and coherent scattering, and calculating the parameters of each term to fit the theoretical value. For example, Ohno et al. [20] used two-PFM to calculate the effective atomic number and electron density from multiple CT images. However, because the current two-PFM ignores energy dependence while approximating the fitting parameter from the theoretical value, the method itself has a large error [3].

In this study, we present the inherent error of the aforementioned PFMs that fundamentally affect the results of the calculation of the effective atomic number and electron density. This study proposes a new formalism: a three-PFM, which can be used in multiple spectral CT. Both two- and three-PFMs were used to calculate the effective atomic number and electron density from multiple CT numbers using a photon-counting CT system. A semi-empirical correction method that corrects the differences between the CT values from the photon-counting CT images and the theoretical values in each spectral region was also introduced. The *I*-value was calculated via parameterisation with the effective atomic number, after which the SPR was calculated from the calculated electron density and the *I*-value. Then, the SPRs from both methods were compared with their corresponding theoretical values. To estimate the noise level of the CT numbers obtained from the photon-counting CT, CT numbers, including noise, were simulated to evaluate the robustness of the two- and three-PFMs.

## 2. Materials and Methods

### 2.1. Effective Atomic Number and Electron Density from the Linear Attenuation Coefficient

The X-ray linear attenuation coefficient μ can be related to the atomic cross-section as a function of energy E and a single element Z [1,2]

(1) $$\mu \left(E,Z\right)=\;\rho N_{A}\frac{1}{A}\left\{\sigma^{PE}\left(E,Z\right)+\sigma^{KN}\left(E,Z\right)+\sigma^{SCA}\left(E,Z\right)\right\},$$

where $\sigma^{PE}$ and $\sigma^{KN}$ are the photoelectric and Klein–Nishina cross sections, respectively; $\sigma^{SCA}$ is the correction term for the coherence and binding energy effects; and ρ, $N_{A}$, and A are the mass density, Avogadro’s number, and atomic mass, respectively. Each cross-section term can then be approximated via the PFM [1]

(2) $$\mu \left(E,Z\right)=\;\rho_{e}\left\{K^{PE}\left(E\right)Z^{m}+K^{KN}\left(E\right)+K^{SCA}\left(E\right)Z^{n}\right\},$$

where $\rho_{e}$ is the electron density, defined by $\rho N_{A}\frac{Z}{A}$, and $K^{PE}$, $K^{KN}$, and $K^{SCA}$ are coefficients for the photoelectric and Klein–Nishina terms and for the coherence and binding energy effects term, respectively. These terms can be determined via least-square fits to NIST attenuation coefficients from the NIST XCOM photon cross sections database [21]. Rutherford et al. [1] proposed values for the exponents m and n at 3.62 and 1.86, respectively. For mixtures, the electron density is calculated using Equation (3), and Z is represented by the effective atomic number ($\bar{Z}$) [22]

(3) $$\rho_{e}\;=\;\rho N_{A}\sum_{i}\omega_{i}\frac{Z_{i}}{A_{i}}\;,$$

(4) $$\bar{Z}=\;\sqrt[{\alpha }]{\sum_{i}\omega_{i}Z_{i}^{\alpha }},$$

where $\omega_{i}$ is the fractional weight (i.e., $\sum_{i}\omega_{i}=1$). In general, α in Equation (4) is assigned values between 2.94 and 4 because it varies depending on the attenuation process and the materials being attenuated [22,23]. In our study, we adopted an α value of 3.3 for the theoretical calculations in Section 2.4.5, referring to Landry et al.’s [24] results [3,9,24].

#### 2.1.1. Two-Parameter-Fitting Method

**Figure 1:**
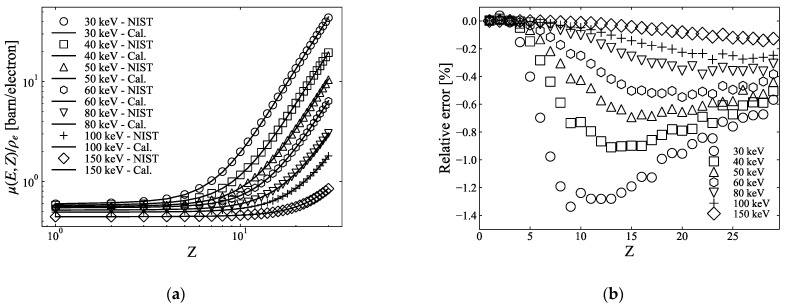
(**a**) $\mu \left(E,Z\right)/\rho_{e}$ (solid line) from 30 keV to 150 keV of atomic numbers up to 29, calculated via two-parameter fitting methods (PFMs) using Equation (5), and corresponding NIST values (marker), (**b**) relative errors between calculated and NIST values in (**a**).

Torikoshi et al. [19] proposed an expression of the linear attenuation coefficient as a photoelectric term and a scattering term based on Jackson and Hawkes’ formulation [2]

(5) $$\mu \left(E,Z\right)=\;\rho_{e}\left[Z^{4}F\left(E,\;Z\right)\;+\;G\left(E,\;Z\right)\right],$$

where $\rho_{e}Z^{4}F\left(E,Z\right)$ is the photoelectric attenuation, and $\rho_{e}G\left(E,Z\right)$ is the combined Compton and coherent scattering term. Yang et al. [3] derived the F(E, Z) and G(E, Z) terms from fitting to attenuation cross-sections, referred from the NIST XCOM database, for each Z in a quadratic equation and interpolating over the entire Z range for several energies. In this study, we employed the method expressed in Equation (5) and referred to it as two-PFM. Figure 1a shows the $\mu \left(E,Z\right)/\rho_{e}$ calculated using Equation (5) from 30 keV to 150 keV obtained using the NIST XCOM database, and their corresponding NIST values and Figure 1b shows the relative errors. The functions F(E, Z) and G(E, Z) are obtained via quadratic fits of the photoelectric and scattering terms of the NIST attenuation coefficients.

Equation (5) is a valid equation for a monochromatic X-ray beam. Using continuum energy spectrum X-rays, we adopted an average attenuation coefficient, which is approximated via integration of the photoelectric and scattering terms weighted with a normalised energy spectrum function [9]:

(6) $${\langle \mu \rangle }_{k}\;=\;\rho_{e}\int_{E_{k}}^{E_{k+1}}\omega_{E_{k}}\left[Z^{4}F\left(E,\;Z\right)\;+\;G\left(E,\;Z\right)\right]dE.$$

The subscript k indicates individual energy threshold levels in the spectral CT: k = 1 to 4, and $\omega_{E_{k}}$ is the weighting function of the spectrum in each threshold

(7) $$\omega_{E_{k}}=\Phi \left(E,E_{max}\;\right)/\int_{E_{k}}^{E_{k+1}}\Phi \left(E,E_{max}\right)dE,$$

where $\Phi \left(E,E_{max}\;\right)$ represents the energy spectrum generated in an X-ray tube with a maximum energy $E_{max}$. From Equation (6), the effective atomic number is calculated from four CT numbers obtained at four energy thresholds using Equation (8)

(8) $${\langle \mu \rangle }_{k}\left[Z^{4}\langle F(E_{k+1},\;Z)\rangle +\langle G\left(E_{k+1},\;Z\right)\rangle \right]-{\langle \mu \rangle }_{k+1}\left[Z^{4}\langle F\left(E_{k},\;Z\right)\rangle +\langle G\left(E_{k},\;Z\right)\rangle \right]\;=\;0,\;\;\left(k=Th1\;to\;Th3\right).$$

where k represents each of the four energy threshold levels in the spectral CT; and $\langle F\left(E_{k},\;Z\right)\rangle$ and $\langle G\left(E_{k},\;Z\right)\rangle$ are the weighted sums of the spectra for F(E, Z) and G(E, Z), respectively. The electron density is then calculated using Equation (9), using the effective atomic number calculated in Equation (8):

(9) $$\langle F\left(E_{k+1},\;Z\right)\rangle \left({\langle \mu \rangle }_{k}-\rho_{e}\langle G\left(E_{k},\;Z\right)\rangle \right)-\langle F\left(E_{k},\;Z\right)\rangle \left({\langle \mu \rangle }_{k+2}-\rho_{e}\langle G\left(E_{k+2},\;Z\right)\rangle \right)\;=\;0,\;\;\left(k=Th1\;to\;Th3\right).$$

The Appendix A details the derivation process for Equations (8) and (9).

#### 2.1.2. Three-Parameter-Fitting Method

**Figure 2:**
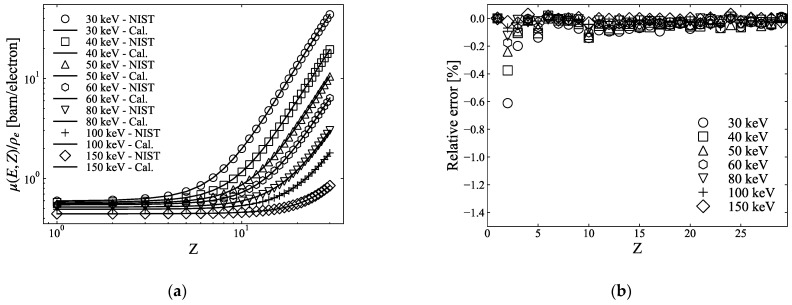
(**a**) $\mu \left(E,Z\right)/\rho_{e}$ (solid line) from 30 keV to 150 keV of atomic numbers up to 29, calculated via three-PFM using Equation (10), and corresponding NIST values (marker), (**b**) relative errors between calculated and NIST values in (**a**).

In our study, we improved the accuracy of $\mu \left(E,Z\right)/\rho_{e}$ by adding energy and atomic number dependency to each index of Equation (2)

(10) $$\mu \left(E,Z\right)=\;\rho_{e}\left\{K^{PE}\left(E,Z\right)Z^{PE\left(E,Z\right)}+K^{INC}\left(E,Z\right)Z^{INC\left(E,Z\right)}+K^{COH}\left(E,Z\right)Z^{COH\left(E,Z\right)}\right\},$$

where $K^{PE}\left(E,Z\right)Z^{PE\left(E,Z\right)}$, $K^{INC}\left(E,Z\right)Z^{INC\left(E,Z\right)}$, and $K^{COH}\left(E,Z\right)Z^{COH\left(E,Z\right)}$ are terms for photoelectric absorption and incoherent and coherent scattering, respectively. In this model, each term has a new dependence on E and Z to improve the approximation accuracy more than that in Equation (2). Hereafter, we refer to this method as three-PFM. Figure 2a shows the $\mu \left(E,Z\right)/\rho_{e}$ calculated using Equation (10) from 30 keV to 150 keV obtained using the NIST XCOM database, and their corresponding NIST values and Figure 2b shows the relative error values. We can see that, compared to two-PFM, as shown in Figure 1b, three-PFM can further reduce the relative errors with respect to the theoretical values.

Equation (10) can be approximated for polychromatic X-rays in the same manner as that used for Equation (6):

(11) $${\langle \mu \rangle }_{k}\;=\;\rho_{e}\int_{E_{k}}^{E_{k+1}}\omega_{E_{k}}\left\{K^{PE}\left(E,Z\right)Z^{PE\left(E,Z\right)}+K^{INC}\left(E,Z\right)Z^{INC\left(E,Z\right)}+K^{COH}\left(E,Z\right)Z^{COH\left(E,Z\right)}\right\}dE.$$

From Equation (11), the effective atomic number can be numerically calculated from four CT numbers obtained at four energy thresholds, as follows:

(12) $$-{\langle \mu \rangle }_{Th2}\sigma_{E_{Th1}}^{Weight}+\left({\langle \mu \rangle }_{Th1}-{\langle \mu \rangle }_{Th3}\right)\sigma_{E_{Th2}}^{Weight}+\left({\langle \mu \rangle }_{Th2}-{\langle \mu \rangle }_{Th4}\right)\sigma_{E_{Th3}}^{Weight}+\;{\langle \mu \rangle }_{Th3}\sigma_{E_{Th4}}^{Weight}=\;0.$$

The Appendix A details the derivation process for Equation (12). $\sigma_{E_{k=1\;to\;4}}^{Weight}$ in Equation (12) represents the sum of the three terms in parentheses in Equation (11) at each threshold k, where each coefficient and exponent are weighted by the spectrum. The electron density was then calculated as follows:

(13) $$\rho_{e}=\frac{\sum_{k}\frac{{\langle \mu \rangle }_{k}}{\sigma_{E_{k}}^{Weight}\left(\bar{Z}\right)}}{4}.$$

#### 2.1.3. Errors of the Two- and Three-PFM

**Figure 3:**
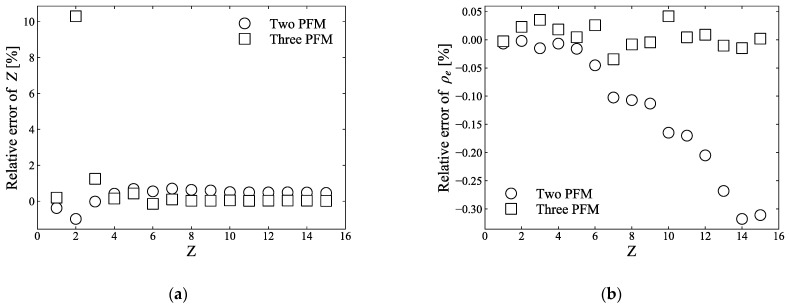
(**a**) shows the relative errors of the atomic number calculated via two-PFM using Equation (8) and three-PFM using Equation (12), respectively, compared to the theoretical values, and (**b**) shows the relative errors of the electron density calculated via two-PFM using Equation (9) and three-PFM using Equation (13), respectively, compared to the theoretical values.

Figure 3a,b show the relative errors of the atomic number and electron density calculated using Equations (8) and (9) for the two-PFM and calculated using Equations (12) and (13) for the three-PFM, respectively, compared to the theoretical values. The calculations were performed under the assumption of monochromatic X-rays, for example, using values of 40, 60, 80, and 100 keV, which are the energies used in the photon-counting CT in Section 2.3. For the two-PFM, the calculated effective atomic number and electron density had relative errors within 1% and 0.35%, respectively, whereas, for the three-PFM, the calculated effective atomic number and electron density have relative errors, based on the theoretical values, within 0.5%, except for atomic numbers 2 and 3 (further discussed in Section 4), and 0.05%, respectively. However, because the effective atomic number of human tissue is between 6 and 15 [4], the errors for atomic numbers 2 and 3 will have a negligible effect on the calculation.

### 2.2. Stopping-Power Ratio from I-Value Parameterisation

The SPR is a parameter used to determine the Bragg curve’s position in ion beam dose calculation and can be calculated using the Bethe–Bloch formula [25], which can be approximated as follows [3,26]

(14) $$\mathrm{SPR}=\frac{\rho_{e,m}}{\rho_{e,w}}\times \frac{ln\left[2m_{e}c^{2}\beta^{2}/I_{m}\left(1-\beta^{2}\right)\right]-\beta^{2}}{ln\left[2m_{e}c^{2}\beta^{2}/I_{w}\left(1-\beta^{2}\right)\right]-\beta^{2}}\;,$$

where $\rho_{e,m}$ and $\rho_{e,w}$ are the electron densities of the medium and water, respectively; $m_{e}c^{2}$ is the equivalent energy of the electron rest mass; $I_{m}$ and $I_{w}$ are the *I*-values of the medium and water, respectively, and β is the ratio of the ion velocity to the speed of light. The *I*-value of the mixture can be calculated using the Bragg additivity rule [27], as follows:

(15) $$lnI_{m}=\;\frac{\sum \frac{\omega_{i}Z_{i}}{A_{i}}\times lnI_{i}}{\sum \frac{\omega_{i}Z_{i}}{A_{i}}}.$$

In this study, the theoretical *I*-values of the elements were obtained from the Particle Data Group (PDG) database [28], which refers to ICRU Report 37 [29], and those of the mixtures were calculated using Equation (15).

**Figure 4:**
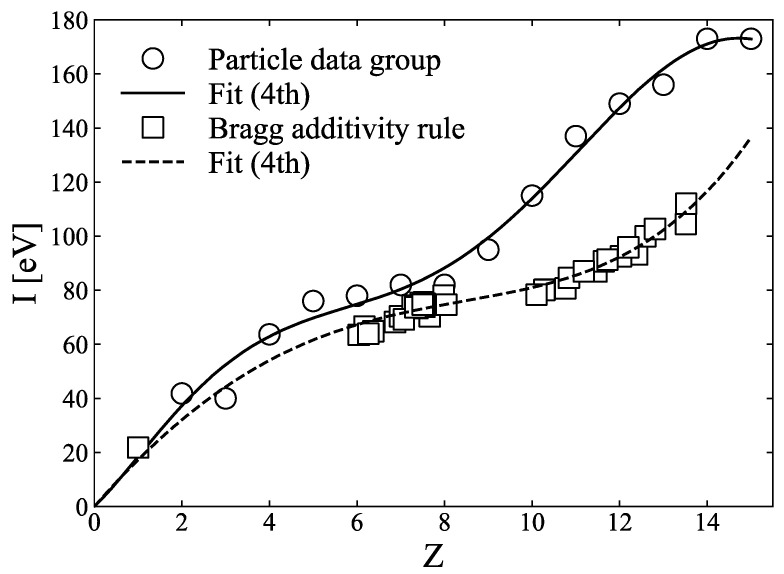
*I*-values of element obtained from the Particle Data Group (PDG) database, and *I*-values of a mixture of human tissue from Table 1 of Bourque et al. [4] calculated using Bragg additivity rule. Each solid line or dashed line is the result of fourth-order polynomial fitting, respectively.

Yang et al. [3] and Bourque et al. [4] have demonstrated that *I*-values can be parameterised as linear or polynomial functions of effective atomic numbers. Figure 4 shows the *I*-value for each element from the PDG and the *I*-values for human tissues, for which we refer to the effective atomic numbers and *I*-values in Table 1 of Bourque et al. [4], calculated using the Bragg additivity rule. The *I*-value for atomic number 1 was added to the mixture value in Figure 4, and least-squares fitting was applied to the fourth-order polynomial of the atomic number, denoted with a dashed line in Figure 4. This fourth-order polynomial fitting result was adopted in the SPR calculation. The *I*-values of the elements, obtained from PDG, were also fitted to the fourth-order polynomial, shown as a solid line in Figure 4.

### 2.3. Photon-Counting CT System

**Figure 5:**
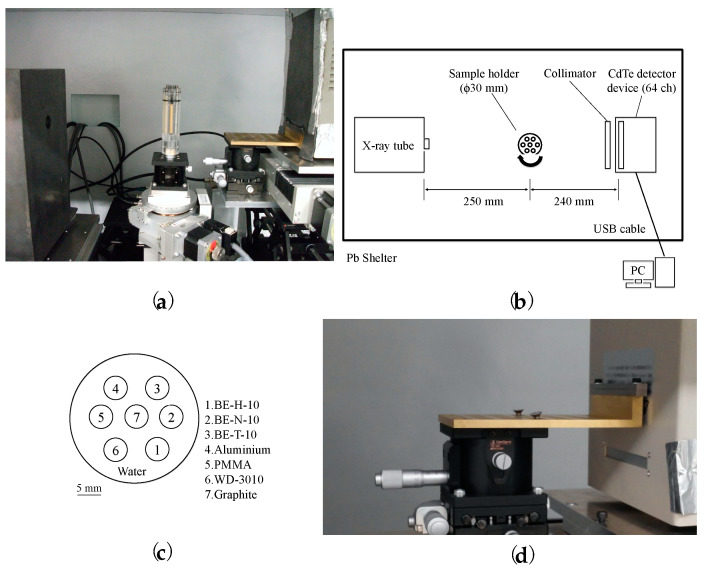
(**a**) picture of the photon-counting computer tomography (CT) system (**b**) schematic view of the photon-counting CT system, (**c**) position of each sample in the sample holder, (**d**) picture of the collimator installed in front of the cadmium telluride (CdTe) detector device.

Figure 5 shows the experimental setup. The photon-counting CT system was housed in a lead-shielded box, which consisted of an X-ray tube with a tungsten target (L12161-07, Hamamatsu, Japan), and included a sample holder, collimator, detector module, and computer. The collimator was made of tungsten with a thickness of 7 mm and 64 circular converging holes, each with a diameter of 0.6 mm and spacings of 1 mm each. This design lowers the transmission of the scattered radiation of 150 keV energy to less than 1% [30]. The sample holder was made of poly(methyl methacrylate) (PMMA) and was cylindrically shaped, 30 mm in diameter, and 85 mm in height, with the capability to rotate 360°. It can hold up to seven samples, each with a 5 mm diameter and 65 mm height. Water fills the space surrounding the sample rods [31]. An energy-differentiation-type 64-channel cadmium telluride (CdTe) radiation line sensor module (C10413, Hamamatsu, Japan) was used as the imaging system. The CdTe detector consists of 64 pixels of CdTe elements, and the size of each element is 0.8 mm (W) × 0.5 mm (H) × 5 mm (T). The elements are aligned with a pitch of 1 mm and a space of 0.2 mm between them. Each CdTe detector was connected to a 64-channel application-specific integrated circuit (ASIC) with 64 low-noise amplifiers. Signal pulses from each detector pixel were differentiated using five comparators according to their energy levels, where each threshold voltage could be controlled by user preference. The measured energy resolution at the full width at half maximum was 10% at 122 keV, which is the main peak of ^57^Co. [32].

### 2.4. Experimental Procedures

#### 2.4.1. Photon-Counting CT Measurement

Seven rod-shaped samples were placed in a water-filled sample holder. The seven materials used for the samples were aluminium, graphite, PMMA, and four types of biological phantom materials (Kyoto Kagaku, Japan), that is, BE-T-10, BE-H-10, BE-N-10, and WD-3010, mimicking the properties of compact bone, cortical bone, internal bone, and water, respectively [31]. For the experimental conditions, the X-ray tube voltage and current were set at 150 kV and 40 μA, respectively, with focal spot sizes of 7 μm. All measurements were performed at room temperature, which was controlled between 20 ± 1 °C. The sample holder rotated 360° at intervals of 2°. The count source period of the detector for each projection was 100 ms and was repeated 100 times to reduce statistical uncertainty, which required 10 s per projection. Each CT number within each energy window was derived by subtracting the data taken above the threshold energy, *k* + 1, from those of *k*: 40 to 60 keV for energy window one, 60 to 80 keV for energy window two, 80 to 100 keV for energy window three, and 100 to 120 keV for energy window four. Each CT image was reconstructed using the fan-beam-based filtered-backprojection method with a Shepp–Logan filter [33], and ring artefacts were removed via a ring correction method in polar coordinates [34] and a three-step correction method introduced by Prell et al. [35].

#### 2.4.2. Spectrum Measurement

**Figure 6:**
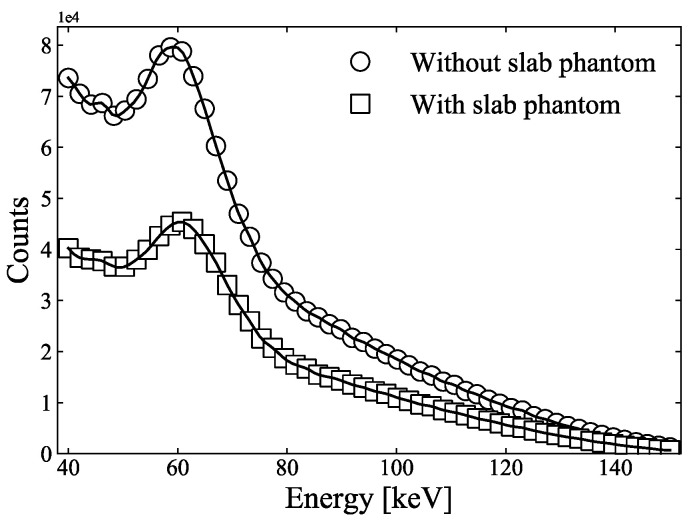
Spectra measured using CdTe line-sensor array, without and with 30 mm-thick slab phantom. Solid lines were calculated using the cubic spline interpolation method.

To obtain the weighted sum from the spectrum to the coefficients and exponent values of each term in Equations (6) and (11), information on the X-ray energy spectrum $\omega_{E_{k}}$ at each threshold energy is required. After determining the weighting sum of each term, we could calculate the atomic number and electron density for the two-PFM and three-PFM. Because the diameter of the sample holder used in the CT measurement was 30 mm, a 30 mm-thick slab phantom was prepared [9] from 15 mm-thick PMMA and 15 mm-thick polypropylene and was attached to the X-ray tube. Fifty-four points (at approximately 2.1 keV intervals) were measured between 40 keV and 150 keV, with the X-ray tube voltage set to 150 kV. Figure 6 shows the spectra, measured using the CdTe line-sensor array, without and with the 30 mm-thick slab phantom. To obtain the spectral weights in Equations (6) and (11), the spectra measured using the 30 mm-thick slab phantom were interpolated using the cubic spline interpolation method to increase the energy resolution. The mean energy at each threshold can be calculated using Equation (16), where Φ(E) is the spectrum obtained from the experiment (Figure 6), and each sum of the spectra were obtained within each energy window.

(16) $${\langle E\rangle }_{k}=\;\frac{\int_{E_{k}}^{E_{k+1}}E\cdot \Phi \left(E\right)dE}{\int_{E_{k}}^{E_{k+1}}\Phi \left(E\right)dE}$$

#### 2.4.3. Semi-Empirical Correction Method for CT Values

**Figure 7:**
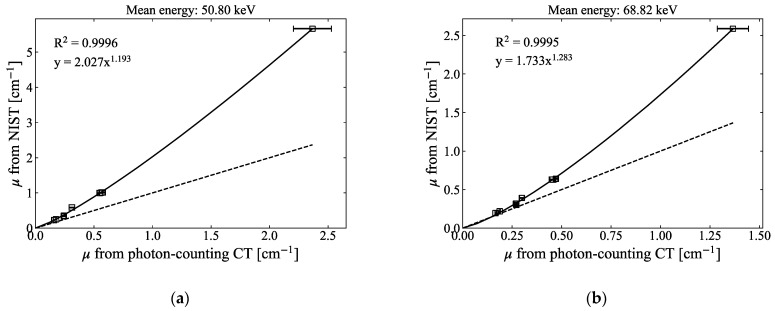

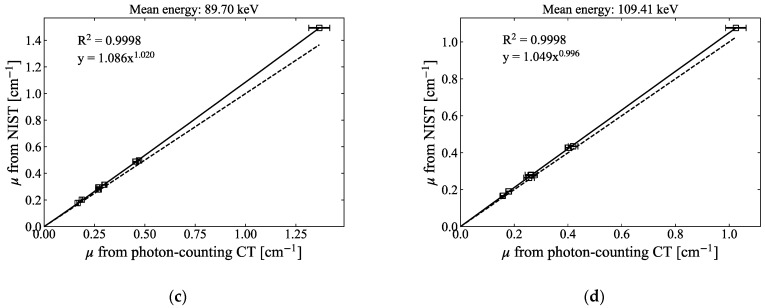
Linear attenuation coefficients obtained from photon-counting CT and those calculated using Equation (17) from NIST values for (**a**) threshold one, (**b**) threshold two, (**c**) threshold three, and (**d**) threshold four. Solid lines are results of fitting to a power function, and broken lines indicate the same values for x- and y-axes (Slope = 1). Mean energy was calculated using Equation (16).

The linear attenuation coefficients derived from the CT numbers using the photon-counting CT system were lower than those of the NIST values. This result can be attributed to the scattered X-rays and inter-pixel charge sharing of the CdTe detector [5,14,36]. Because the coefficients and exponent values of each term in Equations (6) and (11) were determined using the NIST values (by fitting), accurate values cannot be obtained in the effective atomic number and electron density calculations if the linear attenuation coefficients obtained from the CT number are different from the NIST values. Therefore, we applied a correction method that directly compares the linear attenuation coefficient from the CT number with the NIST value. To perform such a correction, each rod sample (5 mm in diameter) of PMMA, graphite, magnesium, aluminium, and titanium was separately placed at the centre of the water-filled sample holder to obtain CT images of each sample with respect to the four thresholds. Each linear attenuation coefficient derived from the CT image was then compared with the NIST value and fitted with a power function. Figure 7 compares the linear attenuation coefficients obtained from photon-counting CT and those calculated using Equation (17) with NIST values for each of the four energy windows.

(17) $${\langle \mu \rangle }_{k}=\;\frac{\int_{E_{k}}^{E_{k+1}}\mu \left(E\right)\cdot \Phi \left(E\right)dE}{\int_{E_{k}}^{E_{k+1}}\Phi \left(E\right)dE}$$

The CT numbers measured in each window were corrected to the expected NIST theoretical value via the plotted power function for each energy window in Figure 7. The corrected CT numbers were then used to calculate the effective atomic number and electron density using the two- and three-PFMs.

#### 2.4.4. SPR Calculation Using Two-PFM and Three-PFM

With the four reconstructed images corrected using the method discussed in Section 2.4.3, we calculated the effective atomic number and electron density of each sample using the two-PFM (Equations (8) and (9)) and three-PFM (Equations (12) and (13)). The *I*-value was then calculated using the calculated effective atomic number, using the parameterisation method shown in Figure 4. Because human tissues, which are mixtures, are actually needed in a clinic, a fitting curve for the fourth-order polynomial fitting of the mixture, calculated using the Bragg additivity rule, was used for the conversion to the *I*-value. For the 290 MeV/u carbon-ion beam, SPR was calculated via the application of the electron density, and the *I*-value derived from the photon-counting CT images to Equation (14). The SPR depends on the energy of ions and has an error within 1% for the energy of the carbon ions used clinically, i.e., from 55 MeV/u to 430 MeV/u. In this study, the energy of 290 MeV/u carbon ions, which will be used to measure the Bragg curve in Section 2.4.5, was utilised for SPR calculation.

#### 2.4.5. Theoretical Values of Effective Atomic Number, Electron Density, I-Value, and SPR

To compare the experimental values for the rod samples used in Section 2.4.1 with their corresponding theoretical values, the theoretical electron density and effective atomic number of the rod samples were calculated using Equations (3) and (4), respectively, and the *I*-values for the elements were obtained from the PDG database, and those for mixtures were calculated using Equation (15). The elemental compositions and mass densities of the biological phantom materials mentioned in Section 2.4.1 were obtained from the catalogue of Kyoto Kagaku [37] and are summarised in Table 1. The method of calculating the SPR by measuring the depth difference of the Bragg curve is conventionally referred to as water-equivalent length (WEL) or water-equivalent path length (WEPL) and has been adopted because of its reliability [38,39]. Here, we adopt the WEL calculation method for the theoretical value of SPR. This method is expressed as Equation (18), where $R_{H,w}$ is the depth of the Bragg curve from the distal H% in only water. $R_{H,m}$ is the depth of the Bragg curve from the distal H% in the water column when a slab material is placed between the water and the ionisation chamber; H is the height from the Bragg peak at the distal portion of the Bragg curve, which is generally represented as a percentage; and $t_{m}$ is the physical thickness of the slab material. In this study, the value of H was 80.

(18) $$\mathrm{SPR}=\;\frac{R_{H,w}-R_{H,m}}{t_{m}}$$

A height-adjustable water column was used to measure the SPR. Depth profiles of the Bragg curves from the 290 MeV/u carbon-ion beam were obtained in water only and for each slab absorber using a plane-parallel chamber (PTW, advanced Markus model 34045) [31]. A slab phantom of each material (aluminium, graphite, PMMA, and four biological phantom materials) was placed between water and an ionisation chamber in a water column. After each Bragg curve was obtained, each SPR was calculated using Equation (18). The values of effective atomic number, electron density, *I*-value, and SPR obtained from photon-counting CT were compared with their theoretical values to calculate their relative errors using Equation (19):

(19) $$\mathrm{Relative}\;\mathrm{error}\;\left[\%\right]=\;\frac{Value_{photon-counting\;CT}-Value_{theory}}{Value_{theory}}\times 100.$$

For the values of the photon-counting CT images, the mean value and standard deviation were calculated by setting the region of interest (ROI) corresponding to the size of each rod sample (5 mm in diameter).

### 2.5. Random Noise Effect for Theoretical Linear Attenuation Coefficient

Compared to single-energy CT or dual-energy CT, photon-counting CT is more susceptible to image noise because only a portion of the photon is counted for each energy bin. Thus, it is necessary to test how the noise in the image affects the results [40,41]. CT numbers, including noise, were simulated to evaluate the robustness of the calculation method described in Section 2.1 and Section 2.2. For noise, a Gaussian distribution was assumed, and a 6% noise level was considered (σ = 6%). Each noise was added to a total of 4096 theoretical linear attenuation coefficients obtained using Equation (17) for each of the seven samples mentioned in Section 2.4.1 with regard to four different thresholds. For 4096 noise-implemented linear attenuation coefficients, the effective atomic number and electron density were calculated for each sample using the two-PFM and three-PFM, and the *I*-value and SPR were calculated using the method described in Section 2.2.

## 3. Results

**Figure 8:**
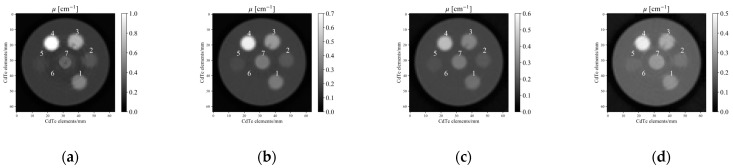
Reconstructed CT images, in which linear attenuation coefficient is corrected via the method in Figure 7, for (**a**) threshold one, (**b**) threshold, (**c**) threshold three, and (**d**) threshold four. The number above the rod in each figure corresponds to Figure 5c: 1. BE-H-10; 2. BE-N-10; 3. BE-T-10; 4. Aluminium; 5. poly(methyl methacrylate) (PMMA); 6. WD-3010; and 7. Graphite.

Figure 8 shows each reconstructed CT image of the seven samples, obtained via photon-counting CT system for the four different thresholds, corrected using the method discussed in Section 2.4.3. Ring artefacts are seen at thresholds one and four because of the high absorption rate at low energy and the low X-ray flux at the detector at high energy. In the case of BE-T-10, the sample itself cracked, and a dent portion occurred in the image.

**Figure 9:**
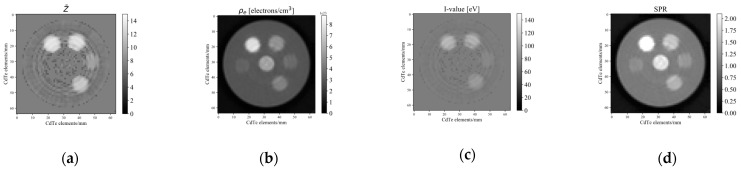
Maps calculated via two-PFM for (**a**) effective atomic number, (**b**) electron density, (**c**) *I*-value, and (**d**) SPR. The *I*-values were parameterised using the fitting curve of the mixture in Figure 4, calculated using the Bragg additivity rule.

**Figure 10:**
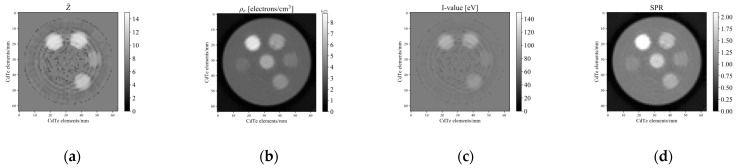
Maps calculated via three-PFM for (**a**) effective atomic number, (**b**) electron density, (**c**) *I*-value, and (**d**) SPR. The *I*-values were parameterised using the fitting curve of the mixture in Figure 4, calculated using the Bragg additivity rule.

Figure 9 and Figure 10 compare the effective atomic numbers, electron densities, *I*-values, and SPRs calculated using two-PFM and three-PFM, respectively, using each of the four images obtained in Figure 8. The ring artefact in Figure 8 is reflected in the calculated image. For the effective atomic number, the centre parts of the sample holder in Figure 9a and Figure 10a have incorrectly calculated parts because of the ring artefacts seen at the centre parts of the images of thresholds 1 and 4 in Figure 8a,d. The *I*-values in Figure 9c and Figure 10c were converted from the effective atomic numbers in Figure 9a and Figure 10a, respectively, via fourth-order polynomial fitting of Figure 4 calculated using the Bragg additivity rule. The SPRs of Figure 9d and Figure 10d were then calculated from electron density (b) and *I*-value (c) of Figure 9 and Figure 10.

**Figure 11:**
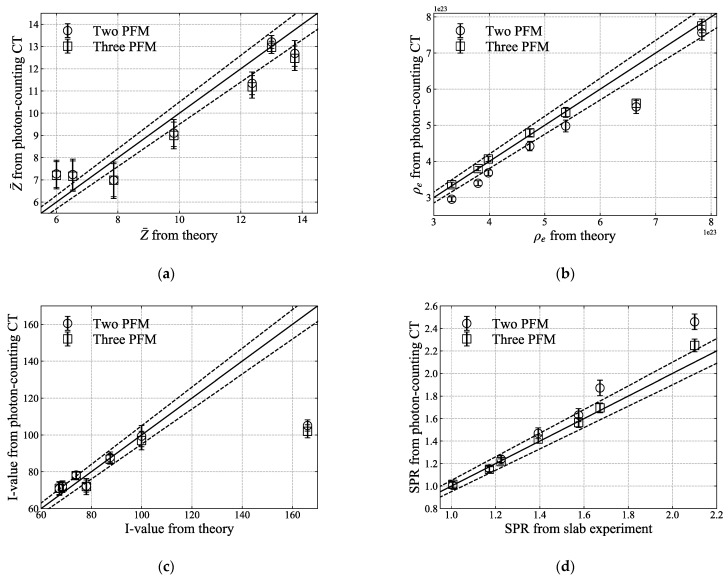
Comparison of (**a**) effective atomic number, (**b**) electron density, (**c**) *I*-value, and (**d**) SPR calculated via two-PFM and three-PFM with theoretical values. Solid lines indicate the same values for x- and y-axes, and the dotted line represents the error level of 5% for the solid line. Standard deviations are plotted together for each value. For the values here, regions of interest (ROIs) were set corresponding to the size of each rod sample in Figure 9 and Figure 10, and the mean and standard deviation were calculated.

**Figure 12:**
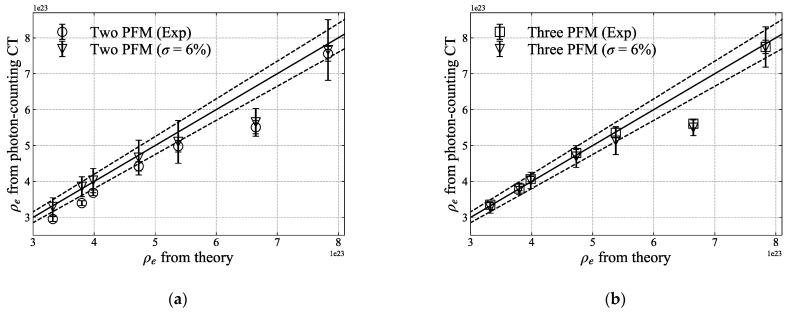

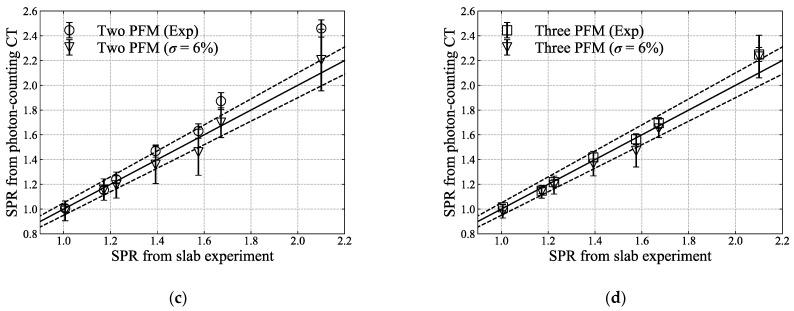
Comparison of experimentally obtained electron density (Figure 11b) and theoretically calculated electron density via (**a**) two-PFM and (**b**) three-PFM, and of experimentally obtained SPR (Figure 11d) and theoretically calculated SPR via (**c**) two-PFM and (**d**) three-PFM. The theoretical values were calculated by adding a 6% noise level to each linear attenuation coefficient from four different thresholds. Solid lines indicate the same values for x- and y-axes, and the dotted line represents the error level of 5% for the solid line. Standard deviations are plotted together for each value.

The values for each of the seven samples are plotted in Figure 11. The error of the SPR in Figure 11d tends to increase as the density of the sample increases. In the case of three-PFM, the overall relative error and standard deviation were less than those of two-PFM. Table 2 summarises the theoretical and experimental values and the relative errors for atomic number, electron density, *I*-value, and SPR. Figure 12 describes the theoretical values of the electron density and SPR in which 6% of noise level were added. As the electron density and SPR obtained from the experiment are within the error range of the theoretical value, it can be estimated that a noise level of about 6% may be included to the CT numbers obtained from the photon-counting CT. For three-PFM, the overall standard deviation was less than those of two-PFM.

## 4. Discussion

Most studies have adopted the two-PFM of Equation (5) for dual-energy CT calculations because it is possible to solve the effective atomic number and electron density clearly when there are two linear attenuation coefficients using two photon energies, assuring a certain degree of accuracy [3,9]. On the other hand, few publications have discussed the accuracy of the fitting method. In contrast, Yang et al. [3] reported that two-PFMs had errors within 1% of the effective atomic number and within 0.35% of the electron density. This result is in good agreement with the calculations shown in Figure 3a,b. Meanwhile, in the conventional approach for the three-PFM using Equation (2) because the exponents m and n of each term are fixed to constants, the calculated $\mu \left(E,Z\right)/\rho_{e}$ has a somewhat large error, depending on the atomic number. We modified Equation (2) for each term to have a dependence on the atomic number, as suggested by Torikoshi et al. [19] for two-PFM, and significantly reduced errors in $\mu \left(E,Z\right)/\rho_{e}$ (Figure 2). Therefore, the errors of the atomic number and electron density calculated using the three-PFM were within 0.5% (except for atomic numbers 2 and 3) and 0.05%, respectively, which were reduced by more than half of those of two-PFM. In Figure 3a, the relative errors of atomic numbers 2 and 3 are fairly high, which seems to be due to the polynomial fitting not fitting well for atomic numbers 2 and 3. For the range of atomic number 1 to 29, compared to the NIST value, atomic number 2 exhibited an error of 8%–10% for the coherent scattering term, and atomic number 3 exhibited an error of 0.6%–0.8% for the photoelectric attenuation. Thus, the relative errors of $\mu \left(E,Z\right)/\rho_{e}$ for atomic numbers 2 and 3 appear to be greater than those for other atomic numbers (Figure 2b). However, because the atomic number of the sample used in this study was between 6 and 15, this error may not have had a significant effect on our calculations. Although both the two- and three-PFMs calculate the electron density after the effective atomic number is calculated, the calculated electron density does not seem to be significantly affected by the calculated effective atomic number (Figure 3).

To calculate the effective atomic number and electron density accurately using both two-PFM and three-PFM, the prerequisite is that the linear attenuation coefficient obtained from the CT number for each threshold should correspond to the NIST value. The linear attenuation coefficient measured using the CdTe detector; on the other hand, did not exhibit the theoretical value at low-energy (50–70 keV) and high-energy regions (120–150 keV). Therefore, the effective atomic number and electron density calculated using the parameters fitted by the NIST values would be lower or higher than the theoretical values. Nakashima et al. [36] and Matsumoto et al. [42] experimented with spectral CT using a CdTe detector and observed that the linear attenuation coefficients measured in low-energy regions were lower than the theoretical values because of scattered X-rays. They installed a molybdenum collimator and reduced the relative error of the linear attenuation coefficient by 16%. We thus fabricated a collimator from tungsten to compare the X-ray spectra and confirmed that the collimator is effective in low-energy regions, as reported by Nakashima et al. [36]. In high-energy regions, on the other hand, the linear attenuation coefficients still differ from the theoretical values. Miyajima [43] reported that the impurities of the detector disturb the drift of charge carriers and cause carrier trapping, which reduces the output pulse height and results in spectrum distortion. They corrected the spectral distortion due to transmission of primary X-rays, escape of secondary X-rays, and tailing, using the stripping method with the detector response function. However, it is considerably difficult to perform all corrections for each 64-detector element for different thresholds and for each material in a single sample holder at the same time. To address these problems, instead of solving the spectrum itself, we chose a semi-empirical method that theoretically corrects the experimental values by directly comparing the CT numbers with the NIST values weighted by the experimental X-ray spectrum for each threshold. In Figure 7, the difference between the theoretical and experimental linear attenuation coefficients increased with increasing material density and was the largest at threshold 1. We experimentally confirmed that the difference between the two linear attenuation coefficients was reduced when the energy width of the threshold was reduced, and we suspect that photon-counting CT was still affected by beam hardening.

In Figure 8, the photon-counting CT images show ring artefacts at threshold 1 (40–60 keV) and threshold 4 (100–120 keV). The artefacts on these images had a direct impact on the calculation of the effective atomic number and electron density. The artefact at threshold 1 seems to be related to the low number of photons because of the large attenuation coefficient, and the artefact at threshold 4 seems to be related to the detector response capable of absorbing high-energy photons. Using Equation (1), proposed by Tsutsui et al. [44], we calculated the X-ray absorbed fraction of our 5 mm-thick CdTe line-sensor array. CdTe absorbs 100% of low-energy photons, but the absorption starts to decrease from 100 keV to 85% at 150 keV. We set the threshold at four, that is, 120 keV, because severe artefacts started appearing on the image from 130 keV.

The effective atomic number calculations for graphite and aluminium demonstrated better results than the relative errors of 35% for graphite and 12% for aluminium in Nakashima et al. [36] and Zou et al. [45], who used two-PFM (Table 2). As shown in Figure 11b, most values of electron density calculated using three-PFM, compared to those of two-PFM, are closer to the theoretical values. The calculation for the SPR image can be considered to be a deformation of the electron density image as much as the influence of the *I*-value map. However, the *I*-value estimation method was different for elements and mixtures (Figure 4). Because we used the *I*-value from the mixture, the relative errors of two-PFM and three-PFM for aluminium were −36.7% and −38.6%, respectively. However, through the use of the *I*-value estimated from the element (fourth-order polynomial fitting), the errors could be reduced to −1.0% and −2.9%, respectively (Table 2). When the SPR is calculated from the element of fourth-order polynomial fitting, the relative errors of two-PFM and three-PFM for aluminium could be reduced by 4% to 12.7% and 2.9%, respectively. The Particle Data Group [28] mentioned that the *I*-value calculation using the Bragg additivity rule could be underestimated because electrons in a mixture are more tightly bound than in free elements. Hiraoka et al. [46] also showed that *I*-values calculated using the Bragg additivity rule tended to be lower than the *I*-values obtained via experimentation, with an uncertainty of ±5%.

A potential disadvantage of multiple-energy imaging is that the signal-to-noise ratio is degraded because the noise increases in proportion to the sum of the squares of the number of images [47]. However, the current study did not consider the evaluation of image quality and noise to see how realistic the SPR calculation results would be with the noise included. Instead, we estimated the noise level included in the reconstructed CT numbers in Figure 8 by adding noise to the theoretically calculated linear attenuation coefficient. As shown in Figure 12, it is thought that the three-PFM will be less affected by the noise contained in the CT numbers than the two-PFM. As Taasti et al. [41] mentioned, this noise level would be expected to be twice that of single-energy CT. We calculated the SPR using a photon-counting CT system, but there seems to be room for improvement in the image-based calculations, such as the inclusion of noise and artefacts of the reconstructed images, to accurately calculate the effective atomic number and electron density.

## 5. Conclusions

The SPR was calculated using a photon-counting CT system after multiple CT numbers were obtained at each threshold. The values for the four CT numbers obtained for the four energy thresholds were calibrated to their corresponding NIST values using a semi-empirical correction method. To calculate the effective atomic number and electron density, we developed a new method, namely, three-PFM, applicable to multiple spectral CT to improve upon the conventional method, namely, two-PFM. Because the three-PFM fits the NIST value more accurately than the two-PFM, the effective atomic number and electron density calculated using the three-PFM exhibited less error than those calculated using the two-PFM. The effective atomic numbers and electron densities of the target materials were derived using each method. The effective atomic numbers were converted into *I*-values via parameterisation. The *I*-values and electron densities were applied to the Bethe–Bloch formula to calculate the SPRs of the target materials. The results of this study demonstrate that three-PFM, compared to two-PFM, calculated SPRs that were closer to the theoretical values and less affected by noise. Therefore, the proposed three-PFM can be potentially used in photon-counting CT, which is more easily affected by noise than single-energy CT or dual-energy CT.

## Figures and Tables

**Table 1:** Elemental compositions and mass densities of the biological phantom materials.

	Elemental Composition (Percentage by Mass)							Mass Density (g cm^−3^)
	H	C	N	O	P	Cl	Ca	
BE-T-10	3.69	29.22	1.19	32.66	10.24	0.06	22.92	1.730
BE-H-10	5.11	42.45	1.73	28.13	7.00	0.09	15.49	1.500
BE-N-10	6.97	60.03	2.45	21.79	2.30	0.13	6.33	1.240
WD-3010	8.63	68.89	2.18	17.88		0.15	2.27	1.018

**Table 2:** Theoretical and experimental values for effective atomic number, electron density, *I*-value, and SPR. Relative error values are indicated in parentheses. Square brackets enclose the experimental values and relative errors when *I*-value and SPR are calculated from the obtained fitting curve using the PDG database in Figure 4.

	Effective Atomic Number			Electron Density			*I*-Value			SPR		
Samples	Theory	Two PFM	Three PFM	Theory (1e23)	Two PFM	Three PFM	Theory	Two PFM	Three PFM	Theory	Two PFM	Three PFM
BE-H-10	12.4	11.3 (−8.3)	11.2 (−9.6)	4.73	4.42 (−6.5)	4.79 (1.2)	87.5	87.8 (0.3)	86.8 (−0.8)	1.39	1.47 (5.5)	1.42 (1.7)
BE-N-10	9.8	9.1 (−7.3)	9.0 (−8.4)	3.98	3.68 (−7.6)	4.07 (2.0)	74.0	78.2 (5.7)	77.9 (5.3)	1.23	1.24 (1.2)	1.22 (−0.6)
BE-T-10	13.8	12.7 (−7.8)	12.5 (−9.4)	5.38	4.98 (−7.4)	5.35 (−0.4)	100.0	99.4 (−0.6)	96.9 (−3.0)	1.58	1.63 (3.5)	1.56 (−0.7)
Aluminium	13.0	13.2 (1.6)	13.0(−0.3)	7.83	7.56 (−3.5)	7.75 (−1.1)	166.0	105.1 (−36.7) [164.4 (−1.0)]	102.0 (−38.6) [161.2 (−2.9)]	2.10	2.46 (17.1) [2.37 (12.7)]	2.25 (7.1) [2.16 (2.9)]
PMMA	6.5	7.2 (10.8)	7.2 (9.8)	3.80	3.40 (−10.6)	3.80 (0.1)	68.5	72.0 (−3.3)	71.7 (−3.6)	1.17	1.15 (−1.6)	1.15 (−2.0)
WD-3010	7.9	7.0 (−11.2)	7.0 (−11.2)	3.32	2.95 (−11.1)	3.35 (0.9)	67.2	70.9 (5.4)	71.0 (5.5)	1.01	1.01 (−0.1)	1.02 (0.8)
Graphite	6.0	7.3 (21.1)	7.2 (20.0)	6.65	5.51 (−17.2)	5.61 (−15.7)	78.0	72.2 (−7.4)	72.0 (−7.8)	1.67	1.87 (12.0)	1.70 (1.4)

## Data Availability

Not applicable.

## Appendix A

We rewrite Equation (6) to Equation (A1)

(A1) $${\langle \mu \rangle }_{k}\;=\;\rho_{e}\left[Z^{4}\langle F\left(E_{k},\;Z\right)\rangle +\langle G\left(E_{k},\;Z\right)\rangle \right],\;\;\left(k=Th1\;to\;Th4\right),$$

where k represents each of the four energy threshold levels in the spectral CT, and $\langle F\left(E_{k},\;Z\right)\rangle$ and $\langle G\left(E_{k},\;Z\right)\rangle$ are weighted sums from the X-ray spectra of Equation (6). If the electron density at each energy threshold is the same, then the aforementioned equation can be written as:

(A2) $$\frac{{\langle \mu \rangle }_{Th1}\;}{Z^{4}\langle F\left(E_{Th1},\;Z\right)\rangle +\langle G\left(E_{Th1},\;Z\right)\rangle }=\frac{{\langle \mu \rangle }_{Th2}\;}{Z^{4}\langle F\left(E_{Th2},\;Z\right)\rangle +\langle G\left(E_{Th2},\;Z\right)\rangle }=\frac{{\langle \mu \rangle }_{Th3}\;}{Z^{4}\langle F\left(E_{Th3},\;Z\right)\rangle +\langle G\left(E_{Th3},\;Z\right)\rangle }\;=\frac{{\langle \mu \rangle }_{Th4}\;}{Z^{4}\langle F\left(E_{Th4},\;Z\right)\rangle +\langle G\left(E_{Th4},\;Z\right)\rangle }.$$

We can then write Equation (A3) by binning each of the two equations of (A2):

(A3) $${\langle \mu \rangle }_{k}\left[Z^{4}\langle F\left(E_{k+1},\;Z\right)\rangle +\langle G\left(E_{k+1},\;Z\right)\rangle \right]-{\langle \mu \rangle }_{k+1}\left[Z^{4}\langle F\left(E_{k},\;Z\right)\rangle +\langle G\left(E_{k},\;Z\right)\rangle \right]\;=\;0,\;\;\left(k=Th1\;to\;Th3\right).$$

Finally, Equation (A3) is summarised with respect to the effective atomic number and becomes Equation (A4):

(A4) $${\bar{Z}}^{4}-\;\left[\frac{{\langle \mu \rangle }_{Th2}\langle G\left(E_{Th1},\;Z\right)\rangle -\sum_{k=Th1}^{Th2}\left\{\left[{\langle \mu \rangle }_{k}-{\langle \mu \rangle }_{k+2}\right]\langle G\left(E_{k+1},\;Z\right)\rangle \right\}-{\langle \mu \rangle }_{Th3}\langle G\left(E_{Th4},\;Z\right)\rangle }{-{\langle \mu \rangle }_{Th2}\langle F\left(E_{Th1},\;Z\right)\rangle +\sum_{k=Th1}^{Th2}\left\{\left[{\langle \mu \rangle }_{k}-{\langle \mu \rangle }_{k+2}\right]\langle F\left(E_{k+1},\;Z\right)\rangle \right\}+{\langle \mu \rangle }_{Th3}\langle F\left(E_{Th4},\;Z\right)\rangle }\right]=0.$$

To calculate the electron density, if the atomic number is the same for each energy threshold, Equation (A1) can be written as:

(A5) $$\frac{{\langle \mu \rangle }_{Th1}-\rho_{e}\langle G\left(E_{Th1},\;Z\right)\rangle }{\rho_{e}\langle F\left(E_{Th1},\;Z\right)\rangle }=\frac{{\langle \mu \rangle }_{Th2}-\rho_{e}\langle G\left(E_{Th2},\;Z\right)\rangle }{\rho_{e}\langle F\left(E_{Th2},\;Z\right)\rangle }=\frac{{\langle \mu \rangle }_{Th3}-\rho_{e}\langle G\left(E_{Th3},\;Z\right)\rangle }{\rho_{e}\langle F\left(E_{Th3},\;Z\right)\rangle }=\frac{{\langle \mu \rangle }_{Th4}-\rho_{e}\langle G\left(E_{Th4},\;Z\right)\rangle }{\rho_{e}\langle F\left(E_{Th4},\;Z\right)\rangle }.$$

In a similar manner, we can write Equation (A6) by binning each of the two equations of (A5):

(A6) $$\langle F\left(E_{k+1},\;Z\right)\rangle \left({\langle \mu \rangle }_{k}-\rho_{e}\langle G\left(E_{k},\;Z\right)\rangle \right)-\langle F\left(E_{k},\;Z\right)\rangle \left({\langle \mu \rangle }_{k+2}-\rho_{e}\langle G\left(E_{k+2},\;Z\right)\rangle \right)\;=\;0,\;\;\left(k=Th1\;to\;Th3\right).$$

We can then summarise Equation (A6) with respect to electron density and write it as Equation (A7):

(A7) $$\rho_{e}=\frac{-{\langle \mu \rangle }_{Th1}\langle F\left(E_{Th2},\;\bar{Z}\right)\rangle +\sum_{k=Th1}^{Th2}\left\{\langle \left[F\left(E_{k},\;\bar{Z}\right)\rangle -\langle F\left(E_{k+2},\;\bar{Z}\right)\rangle \right]{\langle \mu \rangle }_{k+1}\right\}+{\langle \mu \rangle }_{Th4}\langle F\left(E_{Th3},\;\bar{Z}\right)\rangle }{-\langle F\left(E_{Th2},\;\bar{Z}\right)\rangle \langle G\left(E_{Th1},\;\bar{Z}\right)\rangle +\sum_{k=Th1}^{Th2}\left\{\left[\langle F\left(E_{k},\;\bar{Z}\right)\rangle -\langle F\left(E_{k+2},\;\bar{Z}\right)\rangle \right]\langle G\left(E_{k+1},\;\bar{Z}\right)\rangle \right\}+\langle F\left(E_{Th3},\;\bar{Z}\right)\rangle \langle G\left(E_{Th4},\;\bar{Z}\right)\rangle }.$$

In the case of Equation (12), Equation (11) is written as Equation (A8)

(A8) $${\langle \mu \rangle }_{k}\;=\;\rho_{e}\sigma_{E_{k}}^{Weight},\;\;\left(k=Th1\;to\;Th4\right),$$

where $\sigma_{E_{k}}^{Weight}$ is the sum of the three terms in parentheses in Equation (11) at each threshold k, where each coefficient and exponent are weighted by the spectrum. If we assume that the electron density at each energy threshold is the same, then the aforementioned equation can be written as

(A9) $$\frac{{\langle \mu \rangle }_{Th1}\;}{\sigma_{E_{Th1}}^{Weight}}=\frac{{\langle \mu \rangle }_{Th2}\;}{\sigma_{E_{Th2}}^{Weight}}=\frac{{\langle \mu \rangle }_{Th3}\;}{\sigma_{E_{Th3}}^{Weight}}=\frac{{\langle \mu \rangle }_{Th4}\;}{\sigma_{E_{Th4}}^{Weight}}.$$

We can then write Equation (A10) by binning each of the two Equations of (A9):

(A10) $$-{\langle \mu \rangle }_{Th2}\sigma_{E_{Th1}}^{Weight}+\left({\langle \mu \rangle }_{Th1}-{\langle \mu \rangle }_{Th3}\right)\sigma_{E_{Th2}}^{Weight}+\left({\langle \mu \rangle }_{Th2}-{\langle \mu \rangle }_{Th4}\right)\sigma_{E_{Th3}}^{Weight}+\;{\langle \mu \rangle }_{Th3}\sigma_{E_{Th4}}^{Weight}=\;0.$$

Finally, solving the aforementioned equation with respect to $\bar{Z}$ repeatedly yields the solution $\bar{Z}$:

(A11) $$\bar{Z}:\;-{\langle \mu \rangle }_{Th2}\sigma_{E_{Th1}}^{Weight}+\;\sum_{k=Th1}^{Th2}\left\{\left[{\langle \mu \rangle }_{k}-{\langle \mu \rangle }_{k+2}\right]\sigma_{E_{k+1}}^{Weight}\right\}+\;{\langle \mu \rangle }_{Th3}\sigma_{E_{Th4}}^{Weight}=\;0.$$
